# The evaluation of a novel single-lead biopotential device for home sleep testing

**DOI:** 10.1093/sleep/zsae248

**Published:** 2024-10-23

**Authors:** Frederik Massie, Steven Vits, Johan Verbraecken, Jeroen Bergmann

**Affiliations:** Natural Interaction Lab, Thom Building, Department of Engineering, University of Oxford, Oxford, UK; Faculty of Medicine and Health Sciences, University of Antwerp, Antwerp, Belgium; Department of Pulmonary Medicine and Multidisciplinary Sleep Disorders Centre, Antwerp University Hospital, Edegem, Belgium and Research Group LEMP, Faculty of Medicine and Health Sciences, University of Antwerp, Antwerp, Belgium; Natural Interaction Lab, Thom Building, Department of Engineering, University of Oxford, Oxford, UK; Department of Technology and Innovation, University of Southern Denmark, Odense, Denmark

**Keywords:** home sleep testing, biopotentials, sleep staging, deep learning

## Abstract

**Study Objectives:**

This paper reports on the clinical evaluation of the sleep staging performance of a novel single-lead biopotential device.

**Methods:**

One hundred and thirty-three patients suspected of obstructive sleep apnea were included in a multi-site cohort. All patients underwent polysomnography and received the study device, a single-lead biopotential measurement device attached to the forehead. Clinical endpoint parameters were selected to evaluate the device’s ability to determine sleep stages. Finally, the device’s performance was compared to the clinical study results of comparable devices.

**Results:**

Concurrent PSG and study device data were successfully acquired for 106 of the 133 included patients. The results of this study demonstrated significant similarity in overall sleep staging performance (five-stage Cohen’s Kappa of 0.70) to the best-performing reduced-lead biopotential device to which it was compared (five-stage Cohen’s Kappa of 0.73). Contrary to the comparator devices, the study device reported a higher Cohen’s Kappa for rapid eye movement (REM) (0.78) compared to N3 (0.61), which can be explained by its particular measuring electrode placement (diagonally across the lateral cross-section of the eye). This placement was optimized to ensure the polarity of rapid eye movements could be adequately captured, enhancing the capacity to discriminate between N3 and REM sleep when using only a single-lead setup.

**Conclusions:**

The results of this study demonstrate the feasibility of incorporating a single-lead biopotential extension in a reduced-channel home sleep apnea testing setup. Such incorporation could narrow the gap in the functionality of reduced-channel home sleep testing and in-lab polysomnography without compromising the patient’s ease of use and comfort.

**Clinical Trials:**

NCT05035992, A Validation Study of the NightOwl Head Sensor

https://clinicaltrials.gov/ct2/show/NCT05035992

## Graphical Abstract

**Graphical Abstract:**
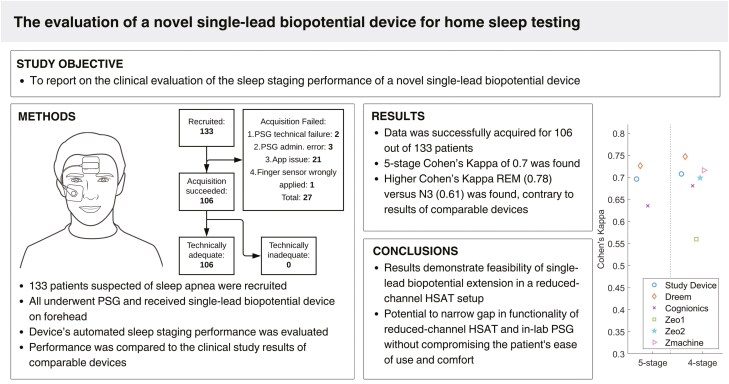

**Statement of Significance**

This paper reports on the clinical evaluation of the sleep staging performance of a novel single-lead biopotential device. The device’s performance was compared to the published clinical study results of comparable devices. Incorporating this device in a reduced-channel home sleep testing setup could widen its applicability, improve its accuracy of sleep stage-derived parameters, and allow for advanced phenotyping of sleep apnea. Such incorporation could narrow the gap in the functionality of reduced-channel home sleep testing and in-lab polysomnography without compromising the patient’s ease of use and comfort.

In-hospital Polysomnography (PSG) has for decades been the gold standard for the evaluation of sleep architecture and common sleep disorders [1]. One of the PSG’s main strengths is its inclusion of a large number of sensing modalities, which allows for a comprehensive assessment of the sleep condition. The PSG setup incorporates multi-channel electroencephalography (EEG), electrooculography (EOG), and electromyography (EMG), from which it can accurately determine sleep stages, thereby classifying sleep into one of the five stages: Wakefulness, rapid eye movement (REM) sleep, and three stages of non-REM sleep (N1, N2, and N3). N1 and N2 are referred to as light sleep, whereas N3 is denominated as deep sleep [2]. Assessing these sleep characteristics and other derivations, such as sleep onset and latencies, is crucial in sleep disorder diagnostics. For example, the diagnosis of obstructive sleep apnea (OSA) relies on the accurate estimation of the total sleep time (TST) to calculate the apnea–hypopnea index (AHI). Furthermore, interest in comprehensive OSA phenotyping for developing personalized OSA management is growing [3]. REM-predominant OSA, for instance, is one phenotype that can inform healthcare providers about choosing an optimal therapy strategy [4].

However, EEG analysis from PSG is not without limitations. It is a costly and time-consuming procedure, requiring highly trained personnel’s assistance for sensor application, patient monitoring, and data scoring. The high density of attached sensors and electrodes combined with an unfamiliar sleep environment may cause discomfort and alter the patient’s natural sleep patterns. Factors such as night-to-night variability and the first-night effect can further decrease the reliability of the PSG analysis [5]. This is why, in recent years, ambulatory versions of the PSG that support in-bedroom, multi-night assessment of sleep have gained popularity. Notably, reduced channel HSAT devices, such as those based on peripheral arterial tone (P-HSAT), are well-positioned to address this need [6]. P-HSAT devices typically consist of a finger-based photoplethysmography (PPG) and accelerometry sensing module from which signal modalities, such as SpO_2_, pulse rate (PR), activity, and peripheral arterial tone, can be derived and analyzed to estimate the AHI. These devices’ lack of EEG, EOG, and EMG sensing capabilities is a significant limitation, prohibiting the conventional and accurate assessment of sleep stages.

Therefore, we have developed an optional extension to a P-HSAT system, which is worn on the forehead and contains a biopotential measurement sensor (from now on referred to as the “study device”). While the study device is anticipated to most frequently operate in conjunction with a P-HSAT system, it can also be used in a standalone fashion. The base of the device, comprising the reference electrode, is attached to the center of the forehead using an adhesive electrode patch to which it is attached via a snap connector. The measurement electrode is connected via an electric wire to the device’s base and is attached below the eye via another adhesive electrode patch (Figure 1). From the biopotential measurement sensor, the software derives single-lead EEG, EOG, and EMG data by filtering the raw biopotential data. The device’s base also contains an accelerometer to determine the head orientation (supine versus non-supine) and a microphone to record nocturnal sound levels.

**Figure 1.**
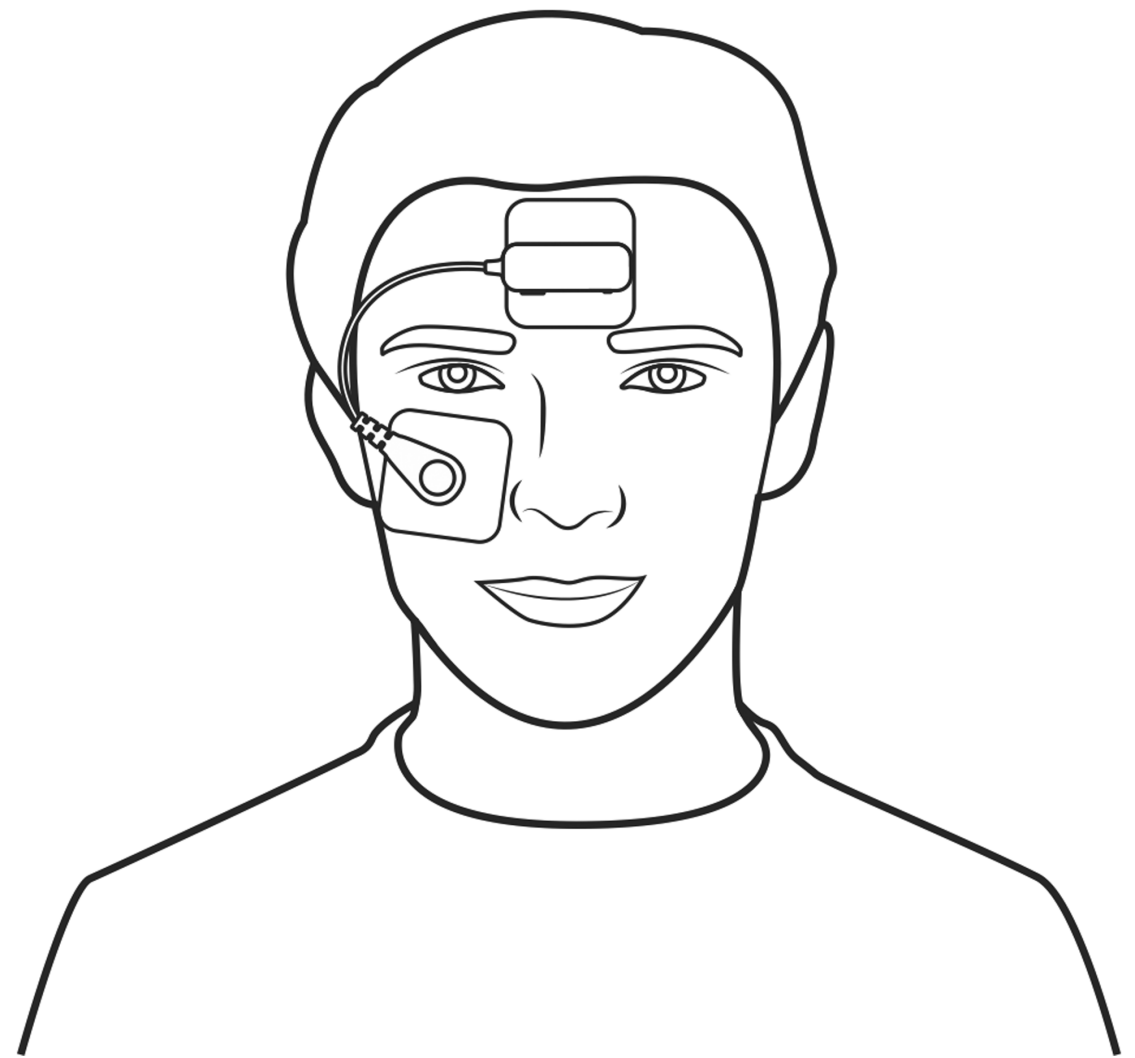
Pictogram of the study device. This pictogram depicts the study device. The reference electrode is attached via an adhesive patch to the center of the forehead. Similarly, the measuring electrode is attached below the eye.

A multi-channel sleep lab EEG setup is usually derived from the standard 10–20 protocol proposed by Herbert Jasper [7]. This system identifies a set of anatomical landmarks to guide electrode placement. Electrodes are placed at 10% or 20% intervals along the anatomical directions with the ground electrode. Different electrodes present distinct sleep signatures depending on their position. The AASM recommends using frontal electrodes to detect K-complexes and delta wave activity, central electrodes to detect sleep spindles, and occipital electrodes to detect alpha waves [2]. These sleep signatures are used to identify sleep stages and are crucial for visual EEG sleep assessment. Since the study device consists of a single-lead biopotential module, which can be considered to record a superposition of frontal EEG, EOG, and EMG, it does not unambiguously present each of the typical sleep signatures. Advanced machine learning techniques, like deep learning methods, can overcome this challenge by learning and interpreting sleep-related patterns that are visually less straightforward to discern. Deep learning networks have some advantages over traditional machine learning algorithms in that they do not require domain knowledge for manual feature extraction and can, therefore, easily self-adapt to different biopotential setups [8]. The study device uses a deep learning network framework consisting of epoch and sequence-based recurrent neural net (RNN) layers. RNNs are particularly effective in capturing temporal dependencies in time series, such as biopotential data [8]. In this paper, we evaluate its performance in sleep staging.

## Study objective

Incorporating a single-lead biopotential extension in an HSAT setup will widen its applicability, improve its TST estimation accuracy, and allow for extended phenotyping of sleep apnea. It is necessary to attain functionality similar to in-lab PSG while optimizing ease of use, comfort, and cost. This study aimed to compare the automated sleep staging detection of the study device to the standard visual sleep scoring of the PSG. To this end, a population with suspected OSA was recruited in a multi-center trial to undergo routine PSG with simultaneous attachment of the study device.

## Methods

### Patients

One hundred thirty-three patients with suspicion of OSA were consecutively included in a cohort across three different centers located in the United States. All centers were part of the United Sleep Diagnostics group in Florida, USA. All patients were scheduled for one overnight in-lab PSG. Patients were asked for informed consent. Aspire IRB, part of the WIRB-Copernicus Group, approved the study Protocol, Patient Information, and Consent Form. Mentally challenged patients or patients below the age of thirteen were excluded from participation in the study. No patients were excluded based on medical conditions or drug treatments. Recruitment occurred between October 5, 2021, and June 2, 2022. For all patients, gender, age, and body mass index (BMI) were recorded. Patients also completed the FDA’s self-completion questionnaire for ethnicity and race.

### Protocol and devices

Routine PSG was performed in all patients. Qualified lab technicians at each participating study center were responsible for setting up the equipment and capturing PSG data. The study device was applied using two adhesive electrode patches: one positioned centrally on the forehead and one below the eye. A snap connector attached the reference and measurement electrodes to these patches (Figure 1). To allow for data synchronization, a P-HSAT (NightOwl, ResMed, USA) was placed on the finger next to the pulse oximeter of the PSG and was time-synchronized via Bluetooth to the study device.

### Polysomnography

The Compumedics E-Series (Compumedics, Australia) PSG was used in all study centers. PSG data was scored centrally by qualified sleep scorers employed by United Sleep Diagnostics, further referred to as the “PSG Analysis.” All PSG data were scored according to the 2020 AASM scoring rules [2].

### Statistical analysis

#### General.

Statistical formulas and calculations were implemented using the MATLAB programming language (version 2019a, MathWorks, USA). The study device was compared to the PSG analysis outcome to compute the endpoint parameters. For all endpoint parameters, 95% confidence intervals were calculated. Confidence intervals were computed for proportion-based endpoints by approximating the error distribution around a binomially distributed observation with a normal distribution. Significance levels were set at a *p*-value of .05.

#### Data synchronization.

Synchronization between the study device and PSG was achieved by shifting and linearly stretching the PR trace of the P-HSAT until the most accurate overlap with the PR trace of the PSG was found. This allowed the synchronization of all other traces since the hypnogram of the PSG is precisely synchronized to the PR trace of the PSG, as they rely on the same hardware clock, and since the P-HSAT and the study device’s Bluetooth data streams are synchronized by design. This method achieved a synchronization error of less than 100 milliseconds. Data epochs of the study device with high lead impedance and Bluetooth disconnections were rejected from the analysis. Epochs from the PSG were rejected whenever the sleep technician deemed the data unreliable for scoring.

#### Data adequacy.

A test was considered technically adequate if at least 30 minutes of overlapping TST between the study device and the PSG could be obtained. This cutoff criterion of technical adequacy was employed to include patients’ data in the dataset for which the endpoint parameters were determined. Other causes for a technical failure might be an early interruption of data acquisition due to acquisition software crashes or other connectivity issues with the data acquisition app. When the PSG recording was technically inadequate, for instance, when one of the channels could not be interpreted by the technicians, the patient was excluded from the analysis. Similarly, when PSG data or any annotations were missing due to administrative errors, the patient was removed from further analysis. Patients with missing patient characteristics, such as age and gender data, were omitted from the study of population demographic statistics but were still included in the endpoint statistics.

#### Performance endpoint selection.

Since there are no standardized performance targets established for sleep stage determination, endpoints that are consistently reported on in other validation studies of wearable biopotential-based sleep staging devices were selected because they facilitate a transparent comparison between those studies. Starting from Imtiaz’s “A Systematic Review of Sensing Technologies for Wearable Sleep Staging” [9], we identified validation studies from peer-reviewed articles on wearable biopotential devices using at least EEG suited for at-home sleep staging analysis. As such, validation studies based solely on biopotential sensing modalities that used a commercially available wearable were included. Studies reporting on automated sleep staging that relied on single-channel data extracted from the PSG itself, such as those proposed by Berthomier et al. [10] and Popovic et al. [11] were excluded. In-ear EEG studies were also excluded due to their fundamental differences from conventional EEG technologies, such as their proximity to reference and measurement electrode placement. Other device studies referenced in the study papers included in Imtiaz’s review that fulfilled these requirements were also included. Finally, the dataset was extended by searching in Google Scholar using the search term “EEG sleep staging validation.” We identified validation studies on six wearable EEG devices:

1. Zeo (Axon Labs, USA), three frontal electrodes [12, 13]
2. Dreem (Beacon Biosignals, USA), five electrodes (O1, O2, FpZ, F7, and F8) [14, 15]
3. SOMNOwatch (SOMNOmedics, Germany), 10 electrodes including EEG, EOG, and EMG [16, 17]
4. SleepProfiler (Advanced Brain Monitoring, USA), three frontal electrodes [18–20]
5. Zmachine (General Sleep, USA), single-lead (A1–A2) [21]
6. Cognionics study device (Cognionics, USA), two-lead (location unspecified) [22]

Informative and transparent endpoint parameters most consistently reported in these studies were stage-wise and overall accuracy, stage-wise and overall Cohen’s Kappa, and stage-wise sensitivity and specificity. Another commonly reported outcome is the sleep staging confusion matrices. When the aforementioned parameters are not readily available, they can be calculated from the confusion matrices if provided in the respective studies. All included studies report on endpoint parameter values derived from the standard 30-second epoch hypnogram. Equally, the study device’s sleep staging endpoint parameter values were derived from its 30-second epoch hypnogram. As sensitivity and specificity are 2-class parameters, they can only be calculated stage-wise. Accuracy and Cohen’s Kappa, on the other hand, can be calculated both on a stage and on an overall level. Cohen’s kappa coefficient (κ) is a statistic used to measure inter-rater reliability for categorical items. It is generally considered a more robust measure than the simple percent agreement calculation, as κ considers the possibility of the agreement occurring by chance [6]. 95% confidence intervals are calculated for each of the agreement measures.

Additionally, we compared parameters that are commonly derived from the sleep stages to the PSG analysis, specifically the TST, sleep efficiency (SE), sleep onset latency (SOL), wake after sleep onset (WASO), and time spent in each of the sleep stages.

The total recording time (TRT) is the total sum of non-rejected epochs, and TST is the sum of those epochs spent in sleep. SE is then calculated as TST/TRT × 100 %. The SOL is the time from the start of the recording until the first three consecutive epochs of sleep. WASO is the time spent in the wake after the SOL point. For each of these parameters and the time spent in each sleep stage (wake, N1, N2, N3, REM), we calculated the mean, bias, and Pearson correlation coefficient, together with their 95% confidence intervals. Bland-Altman analysis plots were used to set out the differences between the PSG versus study device estimates for each parameter while also indicating the mean difference and 95% limits of agreement. It facilitates discussion of underestimation or overestimation biases of the study device and the variability of the accuracy of the study device estimates.

#### Sample size determination.

Statistical power was determined by postulating that the maximum width of the 95% confidence interval of the Cohen’s Kappa of the least prevalent sleep stage (N1) should be at most 0.1. Assuming a baseline Kappa parameter value of 0.3 and an equal rate of false positives and negatives, a minimum sample size of 20 patients was found under the conservative assumption that the average time spent in N1 per patient would be 15 minutes for an average TST of 5 hours.

### Algorithm

#### Data preprocessing.

The raw biopotential data was bandpass filtered between 0.5 and 35 Hz and resampled from 256 to 100 Hz to accelerate the overall processing time. Although the AASM recommends bandpass filter settings with cutoffs at 0.5 and 35 Hz for EEG data and a minimum sampling rate of 200 Hz [2], we found no performance reduction when using our more processing time-optimized alternative settings. Next, each patient’s biopotential trace was normalized by subtracting the median and dividing by the interquartile range. Since the sleep staging model (SSM) makes use of a recurrent neural network (RNN) that expects a two-dimensional matrix as input, the biopotential signal was transformed into a time-frequency image X using a Short-Time Fourier Transform (STFT). As input parameters for the STFT, we have selected a Hamming window with a 50% overlap. The window size was 2 seconds. Logarithmic scaling was used to obtain the log-power spectrum applied: $X_{z}=20\times log_{10}\left|X\right|$. These power spectrograms could then be epoched to serve as input data to the deep learning models.

#### Neural network architecture.

**Figure 2.**
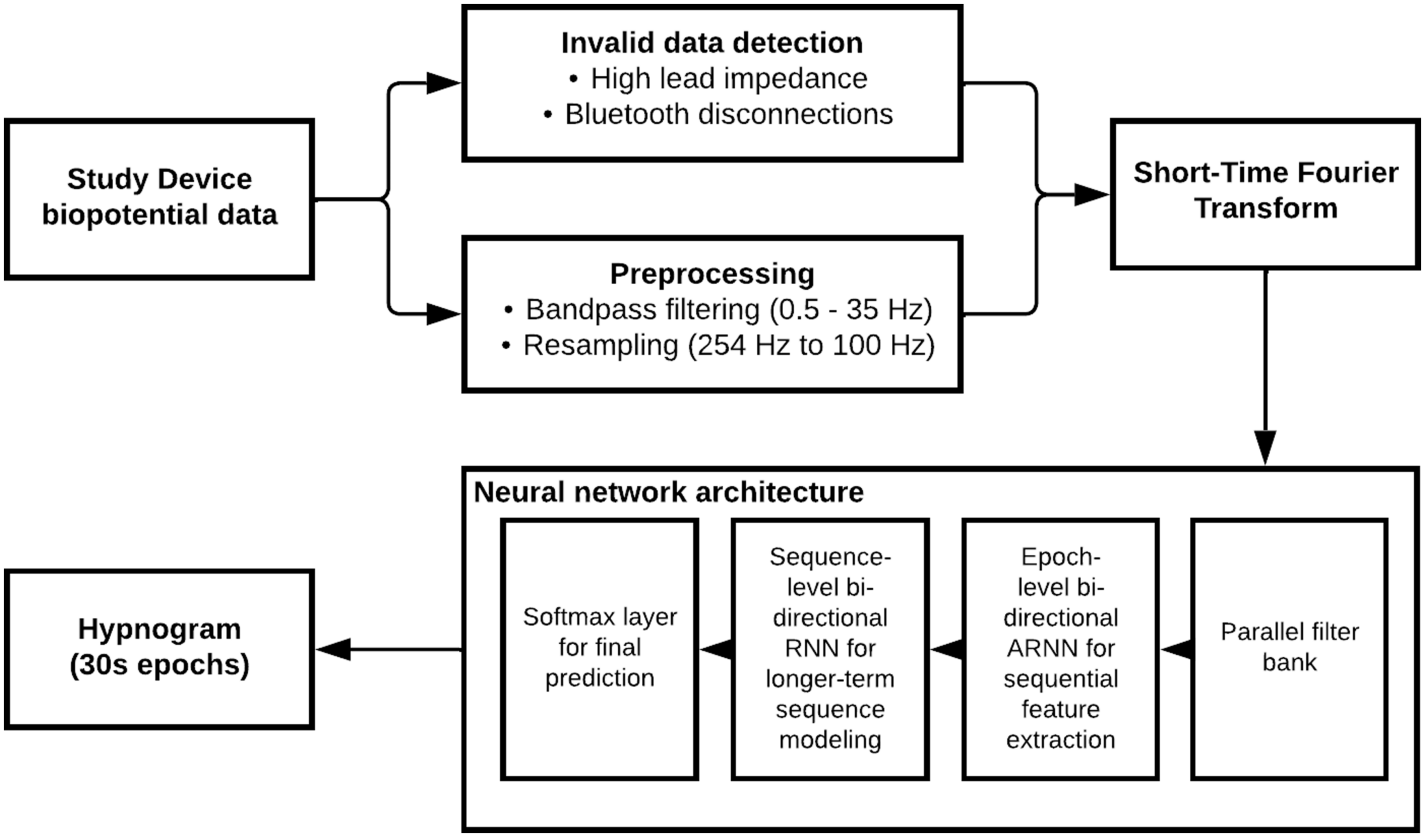
Overview of sleep staging model. This flowchart depicts a high-level overview of the Sleep Staging Model, including the preprocessing steps and neural network architecture.

The central model architecture is an end-to-end hierarchical RNN adapted from Phan et al. [23] Its different layers can be summarized as follows:

1. A parallel filter bank designed to extract the most informative frequency subbands;
2. An epoch-level bidirectional attention-based recurrent neural network (RNN) for sequential feature extraction;
3. A sequence-level bidirectional RNN for longer-term modeling of sequences of epoch-based features;
4. A softmax layer that predicts probabilities for each of the input classes;

This approach enables an end-to-end learning strategy with a global optimization solution while leveraging the sequential characteristics of the sleep data. A high-level overview of the neural network architecture and preprocessing steps is provided in Figure 2.

### Sleep staging model

#### Transfer learning.

Due to the high number of parameters to be trained and the variability in training data, building performant deep neural networks usually requires large amounts of data. Transfer learning solutions can help improve the generalizability and training efficiency of these networks. With transfer learning, the goal is to transfer model knowledge from an already learned source task to the learning of a related target task [24].

To this end, a base model was first trained on a large source dataset. The trained model parameters then served as a starting point for further training and finetuning using the target dataset.

The Sleep Heart Health Study, a multicentric cohort study to investigate cardiovascular and other effects of sleep-disordered breathing, was adopted as the source dataset [25, 26]. The dataset consists of labeled PSG data of 6441 individuals, including C3/M2 and C4/M1 EEGs at 125 Hz. A transfer learning base model using the described neural network architecture is trained on the C3/M2 channel of 200 patients. Base model training input data were extracted by transforming the EEG channel of each patient into its time-frequency spectrum using the SSM settings and dividing the obtained power spectrum into 30-second epochs.

#### Training and cross-validation.

The base model was further finetuned using the target study device dataset. Again, each patient’s preprocessed biopotential channel was transformed into its power spectrum and divided into 30-second epochs. The power spectrum entries of rejected epochs were set to 0, and the corresponding labels were set to “Invalid.” By introducing this additional label, the model automatically learned to deal with rejected epochs while still preserving the data’s sequential nature and time dependence.

To maximize utilization of available data, training and evaluation were performed via a patient-wise 10-fold cross-validation. For each fold, the base model was adapted using 90% of the available patients and was subsequentially evaluated on the remaining 10%. This process was repeated ten times to obtain the total output for each patient independently. This final set of validation outcomes was then used to calculate the endpoint parameters.

## Results

### Patient inclusion and population statistics

**Figure 3.**
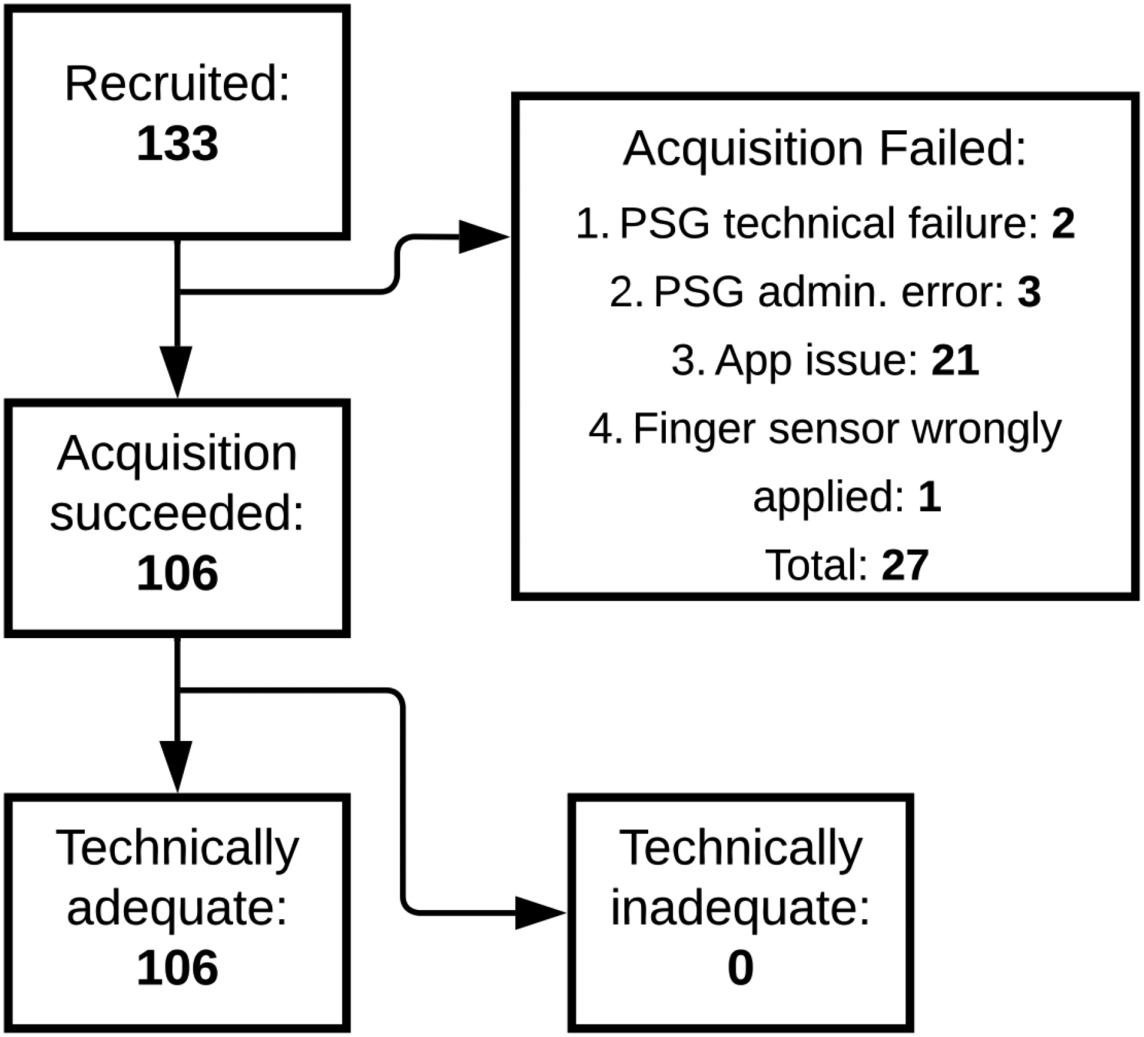
Flowchart of patient recruitment. This flowchart depicts how many patients gave informed consent (133), for how many patients PSG and study device data acquisition was successful (106), and for how many patients the study device data was technically adequate. Additionally, the flowchart summarizes the reasons for data acquisition failure. PSG, polysomnography.

Figure 3 shows the total number of recruited patients and a breakdown of included and excluded patients, together with the reason for failure. Out of the 133 patients who gave informed consent, concurrent PSG and study device data were successfully acquired for 106 patients. For 21 patients, there was an issue with study device data acquisition due to Bluetooth connectivity instability. For two patients, a technical failure of the PSG occurred, resulting in one or more uninterpretable channels. Due to administrative errors, no corresponding match between the PSG and study device recording could be found for three patients. For one patient, the finger sensor device was applied with the optical sensor facing away from the finger, making it impossible to synchronize the data to the PSG recording. The demographic characteristics of the 106 included patients can be found in Table 1. The patients were predominantly male (57%), of middle age (mean 58 years, SD 15), and obese (mean BMI 34, SD 9). The mean AHI was 24 (SD 23). OSA classifications were evenly distributed, with 24% of patients having no OSA, mild OSA, or moderate OSA, and 27% having severe OSA. 28% of patients self-identified as black, 59% as white, and 43% as Hispanic, Latino or Spanish.

**Table 1.** Demographic and Clinical Characteristics of Patients in the Dataset

	Mean	SD	Min	Max
Age	58	15	22	82
BMI	34	9	18	68
AHI	24	23	0	114
	***N* (%)**			
No OSA	26 (24%)			
Mild OSA	26 (24%)			
Moderate OSA	25 (24%)			
Severe OSA	29 (28%)			
Black	30 (28%)			
White	63 (59%)			
Other or no data	13 (13%)			
Hispanic, Latino, or Spanish	46 (43%)			
No data	1 (1%)			

AHI, apnea–hypopnea index, BMI, body mass index, *N*, number of participants, OSA, obstructive sleep apnea, SD, standard deviation.

### Endpoint parameters

**Figure 4.**
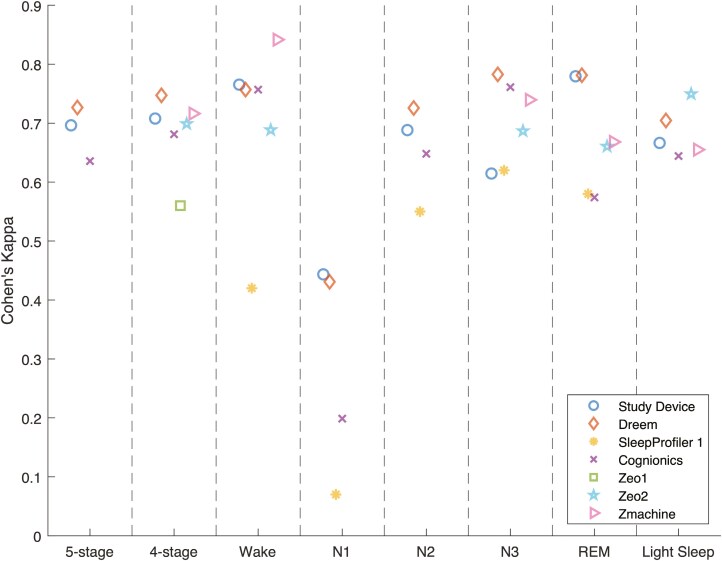
Classification performance plot showing the Cohen’s Kappa for five-stage and four-stage classification and each sleep stage separately. Each marker type represents the same study for the categorizations labeled on the *x*-axis.

Table 2 shows the stage-wise sensitivity, specificity, accuracy, and Cohen’s Kappa for each sleep stage, as well as the overall accuracy and Cohen’s Kappa, including 95% confidence intervals. These endpoints were listed for both the study device and for each of the validation studies found in the performance endpoint selection section. Figure 4 shows the same Cohen’s Kappa values from Table 2 to get a more easily interpretable overview of the devices’ relative performance. A confusion matrix was available from which all parameters were calculated for the Dreem, Cognionics, Zeo 2, and Zmachine studies. Endpoint parameters for SleepProfiler 1 and Zeo 1 were taken directly from the paper when possible. Several studies did not report on N1 and N2 separately but combined these stages into Light Sleep. For easier comparison, the other studies’ overall and Light Sleep agreement parameters were also calculated by combining the N1 and N2 stages. The other stage-wise agreement parameters (wake, N3, and REM) were unaffected. The SleepProfiler 2 study was excluded because the authors arbitrarily combined N2 and N3, making granular comparison to other studies impossible. Both SOMNOwatch studies were excluded because they did not report on stage-wise agreements or present a transparent method for calculating the overall accuracy parameters. Going forward, parameters reporting on all five and four stages with N1 and N2 combined were called five-stage and four-stage classification parameters, respectively.

**Table 2.** Endpoint Parameter Result on Sleep Staging Comparison Summary Table

Device	Stage	Sensitivity	Specificity	Accuracy	Kappa	# Recordings	Ref
Study device	Overall 5-stage	NA	NA	0.797 ± 0.003	0.696 ± 0.003	106	NA
	Overall 4-stage	NA	NA	0.820 ± 0.003	0.708 ± 0.003		
	Wake	0.842 ± 0.005	0.938 ± 0.002	0.915 ± 0.002	0.765 ± 0.003		
	N1	0.472 ± 0.015	0.971 ± 0.001	0.945 ± 0.002	0.444 ± 0.003		
	N2	0.815 ± 0.004	0.875 ± 0.003	0.844 ± 0.003	0.688 ± 0.003		
	N3	0.758 ± 0.011	0.951 ± 0.002	0.936 ± 0.002	0.615 ± 0.003		
	REM	0.795 ± 0.008	0.976 ± 0.001	0.954 ± 0.001	0.780 ± 0.003		
	Light Sleep	0.824 ± 0.004	0.849 ± 0.004	0.835 ± 0.003	0.666 ± 0.003		
Dreem	Overall 5-stage	NA	NA	0.810	0.727	25	[14, 15]
	Overall 4-stage	NA	NA	0.840	0.747		
	Wake	0.776	0.972	0.947	0.757		
	N1	0.476	0.961	0.930	0.431		
	N2	0.827	0.899	0.863	0.726		
	N3	0.878	0.960	0.950	0.783		
	REM	0.860	0.947	0.931	0.781		
	Light Sleep	0.840	0.870	0.853	0.705		
SleepProfiler 1	Overall 5-stage	NA	NA	NA	NA	14	[18]
	Wake	0.442	0.962	0.920	0.420		
	N1	0.270	0.906	0.890	0.070		
	N2	0.718	0.833	0.780	0.550		
	N3	0.603	0.960	0.880	0.620		
	REM	0.724	0.885	0.850	0.580		
Cognionics	Overall 5-stage	NA	NA	0.738	0.636	12	[22]
	Overall 4-stage	NA	NA	0.792	0.681		
	Wake	0.816	0.944	0.915	0.757		
	N1	0.280	0.933	0.887	0.199		
	N2	0.738	0.905	0.826	0.648		
	N3	0.868	0.958	0.948	0.761		
	REM	0.734	0.921	0.899	0.574		
	Light Sleep	0.778	0.874	0.822	0.644		
Zeo 1	Overall 4-stage	NA	NA	0.726	0.560	21	[12]
	Wake	0.408	NA	NA	NA		
	Light sleep	0.800	NA	NA	NA		
	N3	0.621	NA	NA	NA		
	REM	0.737	NA	NA	NA		
Zeo 2	Overall 4-stage	NA	NA	0.811	0.699	26	[13]
	Wake	0.639	0.980	0.929	0.689		
	Light sleep	0.865	0.932	0.919	0.750		
	N3	0.862	0.824	0.845	0.687		
	REM	0.710	0.959	0.930	0.661		
Zmachine	Overall 4-stage	NA	NA	0.822	0.716	99	[21]
	Wake	0.843	0.977	0.948	0.842		
	Light sleep	0.848	0.806	0.829	0.655		
	N3	0.782	0.978	0.963	0.740		
	REM	0.732	0.940	0.904	0.668		

This table displays all endpoint parameter values and their confidence intervals (after the “±” symbol) for the study device, and the included similar devices. Kappa, Cohen’s Kappa; NA, not available.

The study device reported overall Kappas of 0.696 and 0.708 for five-stage and four-stage classification, respectively. The single study that reported statistically significantly higher Kappas for both five-stage and four-stage classification was the Dreem study, with Kappas of 0.727 and 0.747, respectively.

From the stage-wise agreements, it can be observed that N1, consistent with general findings from the scientific literature [27], was the most difficult sleep stage to classify overall, with Kappas ranging from 0.070 (SleepProfiler 1) to 0.444 (study device). As such, the N1 Kappa reported for the study was considerably lower than the Kappa of other stages (of which N3 was the second lowest with a Kappa of 0.615).

Unlike all other studies, the Kappa for N3 (0.615) was significantly lower than the Kappa for REM (0.780).

Across all studies, it could be observed that except for N3 for the Zeo 2 study, the specificity was higher than the sensitivity for all five individual sleep stages. For all studies, the specificity for N1 exceeded 0.9, while the sensitivity ranged from 0.270 (SleepProfiler 1) to 0.4765 (Dreem). This can be explained by the lowest prevalence and, hence, the lowest prior probability of encountering an N1 epoch of all five stages, the typically short duration of N1 stages, and the documented difficulty in scoring N1[27]. As a result, autoscoring systems and human scorers will frequently incorrectly score stage 1 sleep as either wake or N2.

Out of all studies, our study device had the highest statistical power, with 106 successfully included patients, followed by Zmachine, with 99 included patients.

**Figure 5.**
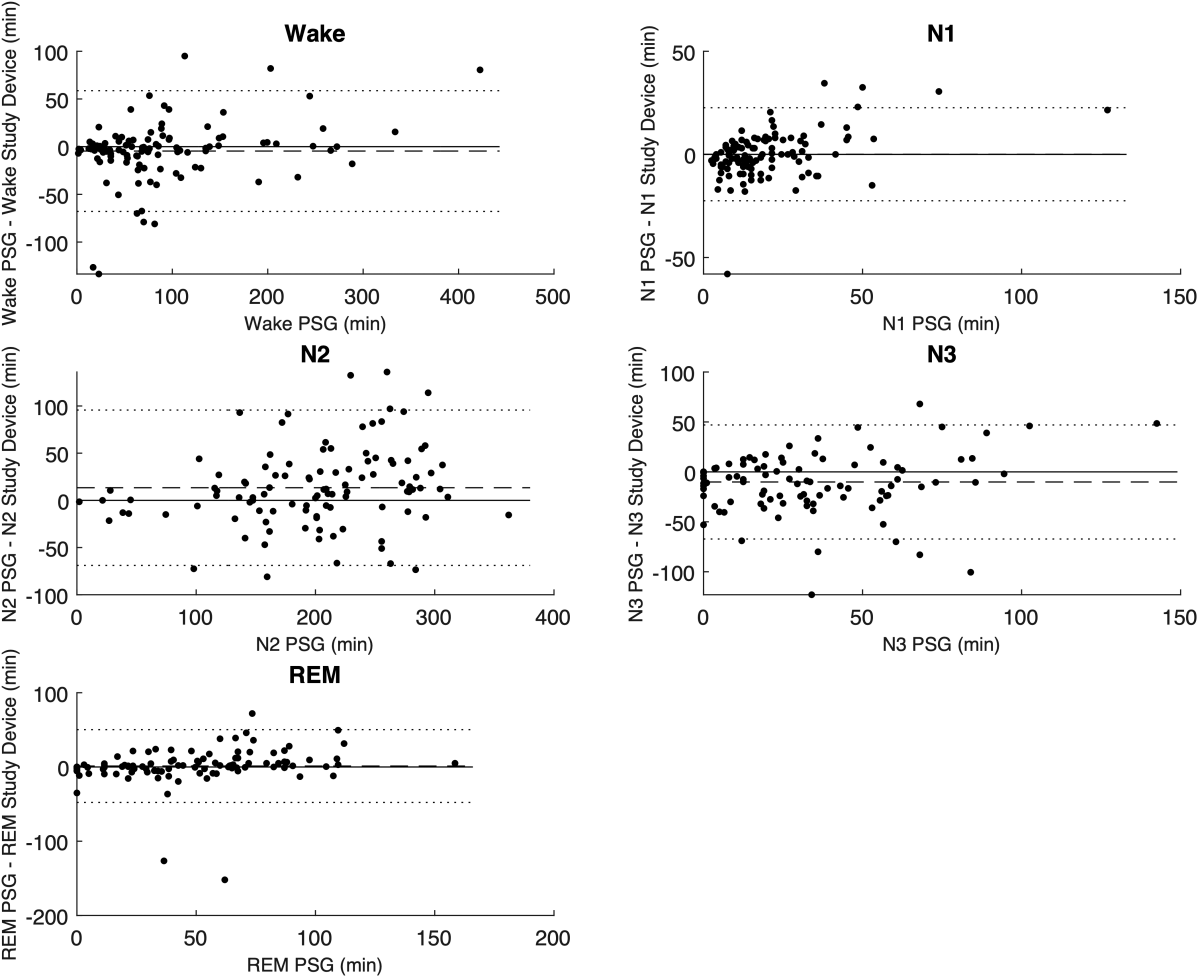
Bland-Altman plots comparing time spent in the five sleep stages between the PSG analysis and the study device. The dotted lines represent the Limits of Agreement. The dashed line represents the mean difference or bias.

**Figure 6.**
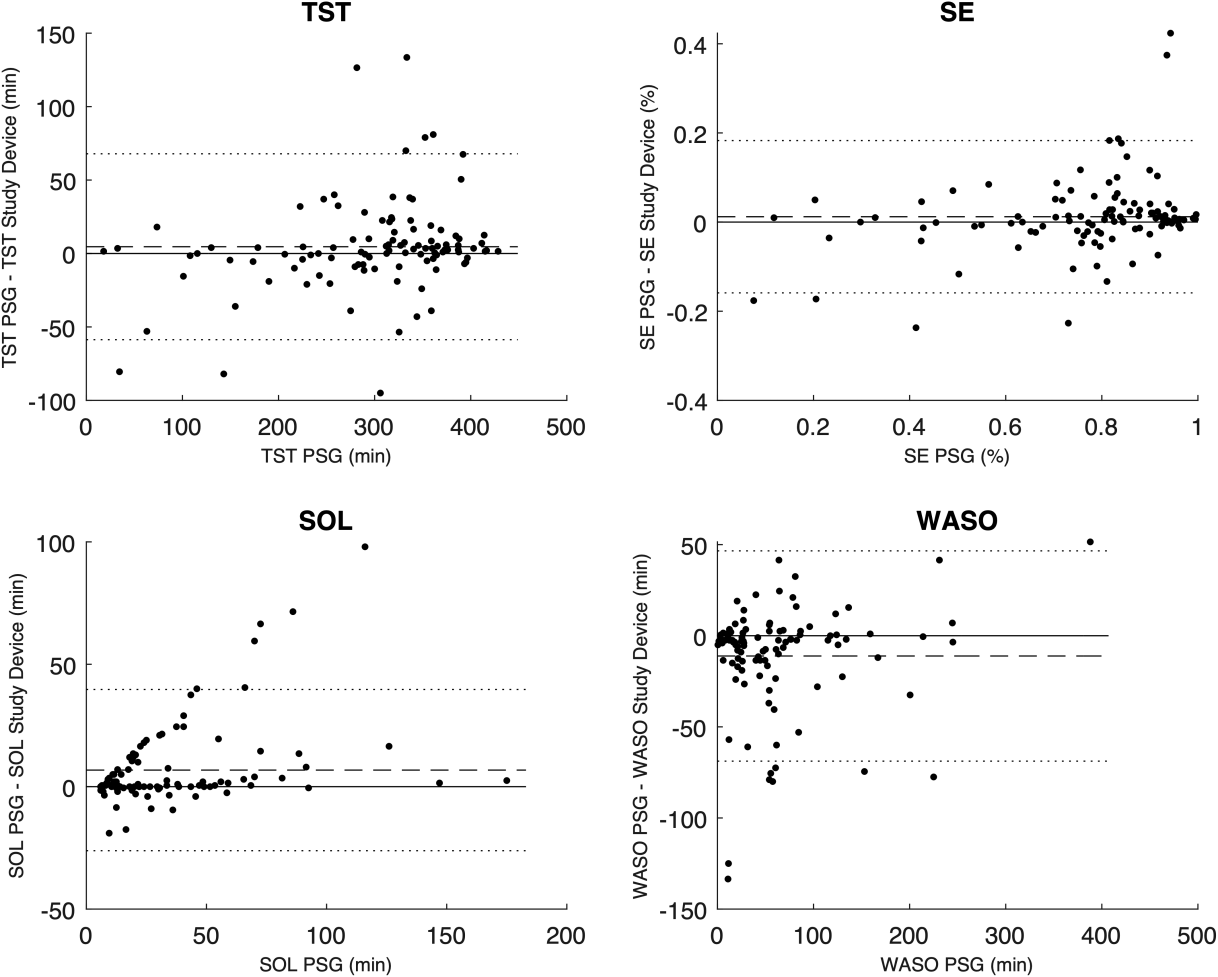
Bland-Altman plots comparing the TST, SE, SOL, and WASO between the PSG analysis and the study device. The dotted lines represent the Limits of Agreement. The dashed line represents the mean difference or bias. TST, total sleep time; SE, sleep efficiency; SOL, sleep onset latency; WASO, wake after sleep onset.

The mean, bias, and Pearson correlation of the aggregated sleep continuity and sleep architecture parameters are set out in Table 3. The results indicate a low bias for each of the estimated parameters, apart from the N1 and N3, which the study device overestimates by about 30% compared to PSG analyses. The correlation coefficient of N3 was markedly lower than for other parameters, which is consistent with the findings from Table 2. The Bland-Altman plots in Figures 5 and 6 give further background to these findings, showing consistent and even dispersion around the mean across the full range of each parameter. The confusion matrix, from which the endpoints in Table 2 were derived, is provided in Table 4.

**Table 3.** Endpoint Parameter Result on Sleep Continuity and Architecture Parameters Summary Table

	PSG mean (min)	Study Device mean (min)	Bias (min)	Pearson ρ
TST	296.09 ± 17.64	291.49 ± 17.07	4.60 ± 6.15	0.938 [0.910; 0.957]
SE	0.762 ± 0.039	0.750 ± 0.037	0.012 ± 0.017	0.905 [0.864; 0.935]
SOL	33.33 ± 5.96	26.55 ± 5.48	6.78 ± 3.20	0.847 [0.783; 0.893]
WASO	62.82 ± 12.32	73.94 ± 12.81	−11.12 ± 5.60	0.901 [0.858; 0.932]
Wake	89.69 ± 14.75	94.29 ± 14.17	−4.60 ± 6.15	0.910 [0.871; 0.938]
N1	20.10 ± 3.21	20.03 ± 2.99	0.07 ± 2.19	0.753 [0.657; 0.825]
N2	199.14 ± 13.86	185.76 ± 14.33	13.38 ± 7.99	0.840 [0.772; 0.888]
N3	30.09 ± 5.51	40.21 ± 7.40	−10.11 ± 5.55	0.667 [0.546; 0.761]
REM	46.75 ± 6.38	45.49 ± 7.10	4.76 ± 0.76	0.755 [0.660; 0.827]

This table displays all endpoint parameter values and their confidence intervals (after the “±” symbol or between []) for the study device and PSG analysis. PSG, polysomnography; TST, total sleep time; SE, sleep efficiency; SOL, sleep onset latency; WASO, wake after sleep onset.

**Table 4.** Sleep Staging Confusion Matrix

		Study device				
		Wake	N1	N2	N3	REM
** PSG**	Wake	**16 017**	1240	1331	57	370
	N1	964	**2010**	1084	18	186
	N2	2329	785	**34 417**	3529	1157
	N3	93	5	1395	**4836**	51
	REM	587	207	1154	84	**7880**

This table displays the confusion matrix comparing the sleep staging category determined by the PSG analysis to the study device. PSG, polysomnography.

## Discussion

### Endpoint analysis highlights and discussion

The most notable discrepancy to the other studies was the study device’s relative outperformance of REM determination compared to N3 since the other studies generally reported a similar performance for N3 and REM or a relative outperformance of N3.

This can be explained by the specific choice of electrode placement of the study device. It has opted to place the electrodes at a diagonal angle across the lateral cross-section of the eye to ensure that the direction of rapid eye movements was readily reflected in the signal as deflections of opposite polarity, which greatly enhances the REM sleep stage detection accuracy and, as was found to be more critical, enhances the capability to differentiate between N3 and REM sleep when using only a single-lead setup. This enhanced differentiation capacity resulted in a marked increase in the five-stage performance compared to alternative single-lead electrode placements. As a result of this optimized single-lead electrode placement, the M1 or M2 (behind the ear) electrode position was sacrificed, resulting in a reduction of the amplitude of delta waves, characteristic of N3, which affects the ability to detect N3. Nevertheless, this reduction in N3 performance in favor of increasing the REM performance was deliberate and desirable as this tradeoff enhances the overall five-stage performance.

The results of our study demonstrate significant similarity in overall sleep staging performance and the tradeoff of specificity and sensitivity for each of the individual sleep stages when compared to the Dreem device, which achieved the highest overall five-stage and four-stage Kappa. In terms of five-stage and four-stage classification performance, the study device ranked third among the seven included studies (with sleep profiler 1 reporting on neither the five-stage or four-stage classification), of which only the Dreem device’s five-stage and four-stage Kappa values were significantly higher. The outperforming Dreem devices make use of five electrodes positioned above the hairline. Our study device’s results are considered favorable, as it was designed as a small form factor with significant consideration for electrode placement to optimize for overall five-stage classification accuracy while minimizing patient discomfort by avoiding electrode placement above the hairline.

### Strengths and limitations of the study

Our study has some strengths and limitations that warrant discussion. The first strength of this study is its adequately powered multicentric design, resulting in a population size of 106 patients. The Zmachine study discussed in this paper reported on 99 recruited patients, whereas the other studies recruited between 12 and 44 patients. The second strength of this study is its inclusion of a comparison of the study device’s sleep staging performance to those obtained from clinical studies of comparable devices.

The first limitation is the inclusion of patients with a suspicion of OSA. While this population matches the primary intended use population for home sleep testing, assessing the device’s performance on a population with a low pretest probability of OSA and a significant prevalence of non-respiratory sleep disorders would be valuable. The second limitation of the study is the relatively large device failure rate due to resolvable instabilities in the Bluetooth connectivity of the prototype study device. The third limitation is the single-scored nature of the reference PSG data. Scoring the PSG data by multiple qualified sleep scorers, ideally from an independent centra, could reveal the inter-scorer agreement on the PSG data to which the study device’s hypnogram can subsequently be compared. Additionally, bundling multiple scorer’s hypnograms, for example, by using a majority voting scheme for each epoch, may serve as a more optimal training dataset, which may further enhance the device’s performance. Finally, like the PSG, the study device relies on EEG, EMG, and EOG data for sleep staging. As the device typically operates as an optional extension to a P-HSAT, it would be valuable to explore whether including peripheral arterial tone, SpO_2_, and actigraphy of the P-HSAT, which are not traditionally used for sleep staging, could further enhance the performance.

## Conclusion

This multi-site clinical evaluation of the study device was designed to robustly assess the device’s capabilities to determine sleep stages automatically. A comparison to similar devices evaluated in clinical studies illustrated that the study device performs similarly to the best-performing device (Dreem), which, in contrast, uses a five-channel setup. This allows us to conclude the feasibility of incorporating a single-lead biopotential extension, which uses a unique performance and comfort-optimized electrode placement, in an HSAT setup. Such inclusion could widen its applicability, improve its accuracy of sleep stage-related parameter determination (such as TST estimation, which would enhance AHI estimation), and allow for extended phenotyping of sleep apnea. Additionally, this improved HSAT setup could narrow the gap in the functionality of reduced-channel home sleep testing and in-lab polysomnography without compromising the patient’s ease of use and comfort.

## Data Availability

The data that support the findings of this study are available from the corresponding author upon reasonable request.
